# Integrative Analysis of Novel Metabolic Subtypes in Pancreatic Cancer Fosters New Prognostic Biomarkers

**DOI:** 10.3389/fonc.2019.00115

**Published:** 2019-02-27

**Authors:** Laura Follia, Giulio Ferrero, Giorgia Mandili, Marco Beccuti, Daniele Giordano, Rosella Spadi, Maria Antonietta Satolli, Andrea Evangelista, Hiroyuki Katayama, Wang Hong, Amin A. Momin, Michela Capello, Samir M. Hanash, Francesco Novelli, Francesca Cordero

**Affiliations:** ^1^Center for Experimental Research and Medical Studies, Azienda Universitaria Ospedaliera Città della Salute e della Scienza di Torino, Turin, Italy; ^2^Department of Molecular Biotechnology and Health Sciences, University of Turin, Turin, Italy; ^3^Department of Computer Sciences, University of Turin, Turin, Italy; ^4^Centro Oncologico Ematologico Subalpino, Azienda Universitaria Ospedaliera Città della Salute e della Scienza di Torino, Turin, Italy; ^5^Servizio di Epidemiologia dei Tumori, Azienda Universitaria Ospedaliera Città della Salute e della Scienza di Torino, Turin, Italy; ^6^Department of Clinical Cancer Prevention Research, MD Anderson Cancer Center, Houston, TX, United States; ^7^Molecular Biotechnology Center, University of Turin, Turin, Italy

**Keywords:** pancreatic cancer, metabolism, cancer subtypes, transcriptomic data, glycolysis

## Abstract

**Background:** Most of the patients with Pancreatic Ductal Adenocarcinoma (PDA) are not eligible for a curative surgical resection. For this reason there is an urgent need for personalized therapies. PDA is the result of complex interactions between tumor molecular profile and metabolites produced by its microenvironment. Despite recent studies identified PDA molecular subtypes, its metabolic classification is still lacking.

**Methods:** We applied an integrative analysis on transcriptomic and genomic data of glycolytic genes in PDA. Data were collected from public datasets and molecular glycolytic subtypes were defined using hierarchical clustering. The grade of purity of the cancer samples was assessed estimating the different amount of stromal and immunological infiltrate among the identified PDA subtypes. Analyses of metabolomic data from a subset of PDA cell lines allowed us to identify the different metabolites produced by the metabolic subtypes. Sera of a cohort of 31 PDA patients were analyzed using Q-TOF mass spectrometer to measure the amount of metabolic circulating proteins present before and after chemotherapy.

**Results:** Our integrative analysis of glycolytic genes identified two glycolytic and two non-glycolytic metabolic PDA subtypes. Glycolytic patients develop disease earlier, have poor prognosis, low immune-infiltrated tumors, and are characterized by a gain in chr12p13 genomic region. This gain results in the over-expression of *GAPDH, TPI1*, and *FOXM1*. PDA cell lines with the gain of chr12p13 are characterized by an higher lipid uptake and sensitivity to drug targeting the fatty acid metabolism. Our sera proteomic analysis confirms that TPI1 serum levels increase in poor prognosis gemcitabine-treated patients.

**Conclusions:** We identify four metabolic PDA subtypes with different prognosis outcomes which may have pivotal role in setting personalized treatments. Moreover, our data suggest TPI1 as putative prognostic PDA biomarker.

## 1. Introduction

Pancreatic Ductal Adenocarcinoma (PDA) is one of the deadliest cancer with patients presenting advanced metastatic disease at diagnosis (1). The reasons of PDA mortality can be attributed to the lack of early symptoms and the absence of diagnostic and prognostic biomarkers (2). Indeed, PDA develops over decades without clinical relevant symptoms and it accumulates mutations in oncogenic drivers (i.e., KRAS) and Copy Number Variations (CNVs). These genomic alterations will reflect in several changes at genes expression level (1, 3).

A hallmark of PDA is the presence of a dense desmoplastic reaction increasing the aggressiveness of this tumor. Indeed, PDA microenvironment is dominated by presence of stromal and inflammatory cells (4). Activated non-neoplastic stromal cells produce extracellular matrix proteins which generate intense interstitial pressure and hypoperfusion which limits oxygen and nutrient availability. The hypoxic regions of PDA tumors have an increased expression level of the lactate exporter monocarboxylate transporter 4 (MCT4). The lactate secreted is used by cancer cells grown in normoxic conditions to proliferate. Moreover, lactate has also effects in the polarization of immunosuppressive macrophages (5–7). Indeed, PDA rewires the neoplastic stromal cell metabolism to maintain its viability and stromal/immune cells interact with cancer cells to metabolically support them. PDA hypovascular nature leads to an increased demand of glucose and aberrant KRAS pathway promote the glucose avidity stimulating the up-regulation of glucose transporter GLUT1, and several other glycolytic enzymes (8–14). Furthermore, an active anaerobic glycolysis characterizes more aggressive and mesenchymal-like tumor PDA subtypes (15, 16).

The PDA molecular classifications have provided new insights in the prediction of the optimal therapy, disease recurrence and in the study of oncogenic genes that lead to metastasis. The advent of *omics* techniques give the opportunity to explore a huge volume of data by inspecting different layers of information ranging from molecular profiles to metabolomic measurements. The majority of classifications uses one layer of data at a time, i.e., gene expression profiles (17–19) or genomic alteration signatures (20), or metabolic data (21). The consideration of data obtained from a single technique is limited, otherwise the integrative use of different *omics* data would be a good method to establish a clinically relevant taxonomy in PDA (22).

Currently, a detailed transcriptomic and genomic analysis of glycolytic subtypes is still missing. A glycolytic addiction of PDA cells was suggested by different authors (23, 24) which observed a strict dependence of the PDA cells proliferation to the glycolytic enzymes overexpression (25, 26). Despite the clear association between aerobic glycolysis and PDA progression, a classification of PDA primary tumors in metabolic subtypes is missing and the molecular drivers of the distinct PDA metabolic subtypes is not sufficiently known.

To tackle this issue, first we integrated transcriptomic and genomic data of The Cancer Genome Atlas (TCGA-PAAD), and International Cancer Genome Consortium (ICGC) patient cohorts. Second, we analyzed transcriptomic and genomic data from PDA cell lines [Cancer Cell Line Encyclopedia, CCLE; (27)], third, we integrated information of metabolomic profiles of PDA cell lines (21). Finally, we performed a pilot proteomic experiment on sera from a cohort of 31 PDA patients to investigate candidate circulating diagnostic biormakers.

We define distinct PDA glycolytic subtypes with different clinical outcomes, Transcription Factors (TFs) expression and sets of recurrent CNVs. We report a recurrent functional gain of chromosome 12 p arm, band 1 sub band 3 (chr12p13) that correlates with glycolytic genes over-expression. By the analysis of transcriptional, metabolic and proteomic data we investigate the effect of this genomic alteration in PDA cell lines and tumors, and we argue that chr12p13 functional gain is a driving genomic alteration of an aggressive PDA metabolic subtype. The clinical role of genes located on chr12p13 as clinical prognostic biomarkers is investigated from our proteomic data. Through this analysis, we identify the glycolytic enzyme TPI1 as a glycolytic biomarker in PDA as its increased level positively correlates with a poor response to chemotherapy (CT).

## 2. Methods

### 2.1. Definition and Characterization of PDA Glycolytic Subtypes

The PDA glycolytic subtypes were defined by RNA-Seq expression analysis of 38 genes coding for glycolytic enzymes. The Z-score-transformed RNA-Seq data from 176 and 99 PDA samples from TCGA PAAD and from ICGC PACA-AU cohorts were analyzed separately. The set of 38 glycolytic genes was defined using Gene Ontology by selecting the GO Term “Glycolytic process” (GO:0006096). Seventy-one genes annotated to this ontological term were isolated using BioMart tool of Ensembl release 86. Among the genes coding for glycolytic enzymes, a subset of 39 genes was selected. Since our study is not focused on glycolysis in sex-specific tissues the genes expressed in testis tissue (*GAPDHS, PGK2*) were excluded from the analysis. Furthermore, since our study includes the catabolism of lactate, which is not included in the glycolytic process, the *LDHB* gene coding for isoform H of *LDH* was included in our list. The clustering algorithm identifies two PDA clusters defined as Glycolytic (Gly) and Non-Glycolytic (Non-Gly) subtypes. Hierarchical clustering was used to define High Glycolytic (HG), Very High Glycolytic (VHG), Low Glycolytic (LG), and Very Low Glycolytic (VLG) subtypes.

Differential analysis of glycolytic genes expression among PDA glycolytic subtypes was performed using Wilcoxon Rank-Sum test, while differential mutation and CNV status analysis was performed using Chi-square test. The *p*-values were corrected using Benjamini-Hochberg (BH) method.

Statistical analysis of clinical data was performed using R (V.3.3.0) and Graph Pad Prism 6. For both TCGA and ICGC, only covariates measured for at least half of patients were considered. *p*-values were computed using Wilcoxon Rank-Sum test for continuous data and Chi-square test for categorical data.

Estimates of the cumulative survival distributions were computed by the Kaplan-Meier method, and the differences among groups were compared using the log-rank test. The median survival of VLG group cannot be computed because survival exceeded 50% at the longest time point. We reported the median survival as “NaN”. The significance of clinical data was also evaluated using the multivariate Cox proportional hazard regression model implemented in the *Coxph* function of *survival* R package. The function was applied with default parameters. Only covariates with at most one NA value were considered.

### 2.2. Evaluation of the Immunological and Stromal Infiltrate

The amount of the immunological and stromal infiltrate among PDA subtypes in TCGA study was evaluated using ESTIMATE (28), by downloading the Stromal and Immunological Scores pre-computed for TCGA-PAAD cohort (PAncreatic ADenocarcinoma, PAAD). The population of tumor-infiltrating immune cells were inferred using TIMER tool at https://cistrome.shinyapps.io/timer. Data of the TCGA-PAAD study were downloaded from the Estimation module (29). Statistical differences among the four subtypes were computed using Wilcoxon Rank-Sum test. TIMER data were used also to evaluate the influence of same samples purity on glycolytic genes expression by retrieving the purity-corrected partial Spearman's correlation and statistical significance provided by the Gene module. The level of immune infiltrate of the TCGA PDAs was evaluated by analyzing the data from Saltz et al. (30). Specifically, considering the data associated with the publication, the percentage of tumor-infiltrating lymphocytes (“til_percentage” parameter) was retrieved for 160 TCGA PDAs out of the 176 samples analyzed in this study.

### 2.3. Differential Genes Expression and Functional Enrichment Analysis

Differentially Expressed (DE) genes among the Gly and Non-Gly and VHG and HG subtypes were identified using *DESeq2* R package (31). Genes associated with an adjusted *p* < 1*E*^−05^ were considered as significantly DE. Functional enrichment analysis was performed using Enrichr (32). Only the top 20 terms associated with adjusted *p* < 0.001 were considered. TF analysis was performed using Enrichr by considering the *ChEA* and *ENCODE* and *ChEA Consensus TFs from ChIP-X* gene set libraries reporting gene set annotated with validated TF-promoter binding. Identification of genes correlated in expression with *FOXM1* was performed using Pearson method. Only genes associated with BH adjusted *p* < 0.001 were considered.

### 2.4. Metabolomic Data Analysis

Metabolomics data of 44 pancreatic cancer cell lines were retrieved from Supplementary Material of (21). Cell lines used in this study were classified as Gained/Amplified or Diploid/Deleted on the basis of their CNV status of *TPI1, GAPDH, ENO2*, and *FOXM1*. Expression and CNV data of these cell lines were retrieved from CCLE. Analysis of metabolic differences among cell line groups was performed by Wilcoxon Rank-Sum test. Analysis of metabolite abundances was performed by considering data from Broad Profiling and Energy platforms.

### 2.5. Study Population of PDA Patients

Mass Spectrometry (MS) analysis was performed on serum samples of 31 patients with PDA not subjected to surgery and treated with gemcitabine-based CT (gemcitabine with oxaliplatin or alone).

The 31 PDA patients were divided in four groups based on their survival and their response to CT. Specifically, the groups 1 and 2 have a median survival >9.86 months while the groups 3 and 4 have a median survival lower than 9.86 months. In the first group patients have disease regression after CT. The second group has stable disease after CT. The third and the fourth groups have progressed disease after CT.

### 2.6. Mass Spectrometry (MS)-Based Proteomic Analyses

Serum samples were isolated from venous blood before CT and at each observation after cycles of CT and stored at –80°C until use. Sera of patients within each group were pooled and 300 μl of serum from each pool were used for MS analysis. Finally, free circulating proteins were isolated after serum depletion of IgG-, IgM-, IgA-bound proteins and HLA-I and -II complexes. Each sample was immuno-depleted, reduced with TCEP, tagged with iodoacetyl tandem mass tag reagents (Thermo), 2D-HPLC fractionated and digested with trypsin as previously described (33). MS analysis was performed by Q-TOF micro (Micromass, Manchester, United Kingdom).

Detailed Materials and Methods are reported in the supplementary text of the manuscript.

## 3. Results

### 3.1. Analysis of Pancreatic Cancer Expression Data Reveals an Aggressive Glycolytic Subtype

**Figure 1:**
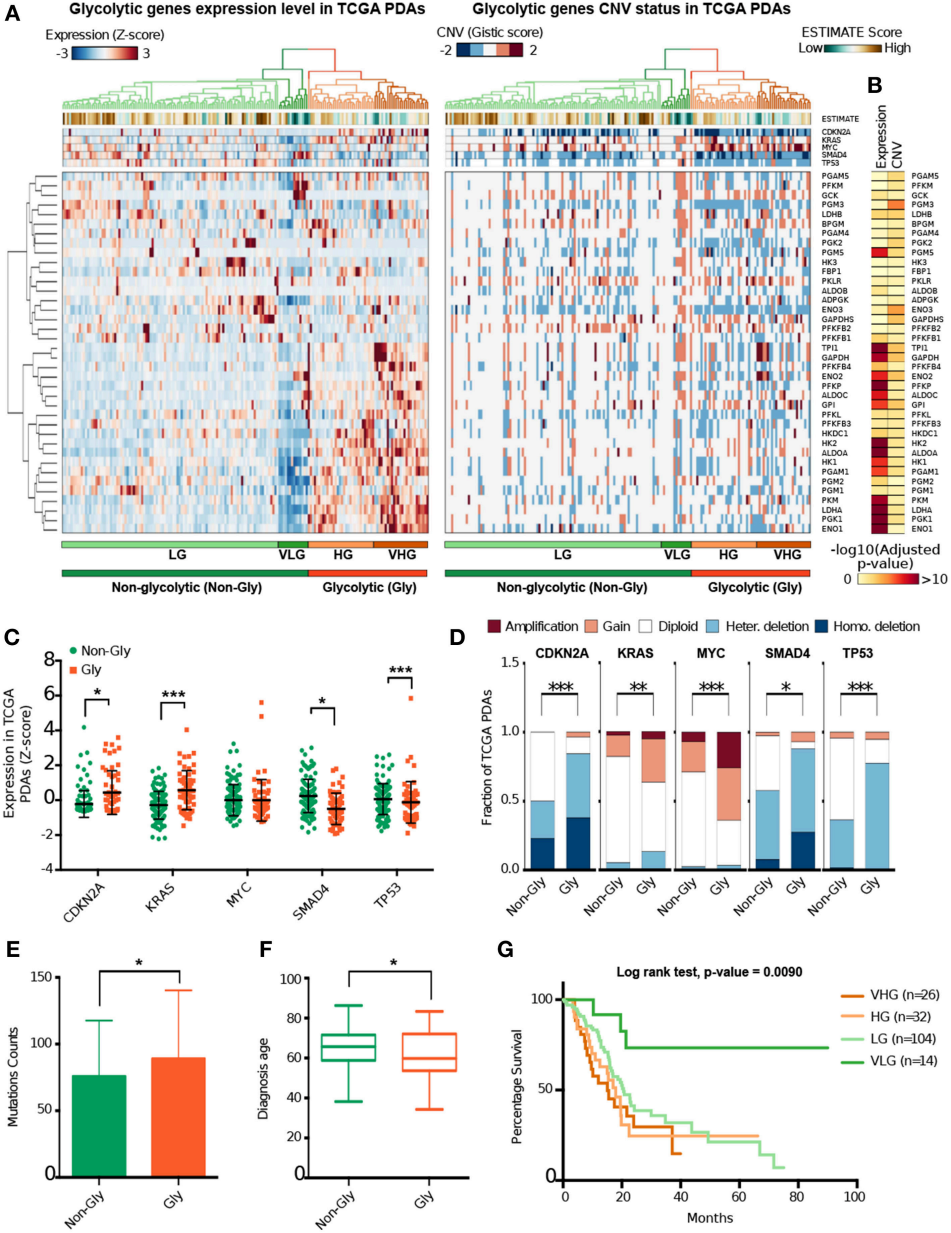
Identification of PDA glycolytic subtypes. **(A)** Heat maps show the normalized level of gene expression (left) and the CNV status (right) of 38 genes coding for glycolytic enzymes in 176 PDA samples from TCGA. At top the expression and the CNV status of five genes involved in PDA tumorigenesis are reported. Green (low score) to brown (high score) colors indicate the ESTIMATE score proportional to the estimated stromal/immune component of each tumor sample. At bottom the main patient clusters are highlighted. Gly, Glycolytic; Non-Gly, Non-Glycolytic, HG, High Glycolytic; VHG, Very High Glycolytic; LG, Low Glycolytic; VLG, Very Low Glycolytic. **(B)** Heat maps reporting the -log10 BH adjusted *p*-value computed between expression level and CNV status of Gly and Non-Gly tumors. **(C)** Dot plot reporting the normalized expression level of five genes involved in PDA tumorigenesis separately for Gly (orange) and Non-Gly (green) PDA patients. *P*-value from Wilcoxon Rank-Sum test. **(D)** Bar plot shows the distribution of CNV events of five genes involved in PDA tumorigenesis separately for Gly and Non-Gly PDA patients. *p*-value from Chi-square test. **(E)** Histogram showing the mutation counts in Gly and Non-Gly PDA patients. *P*-value from Wilcoxon Rank-Sum test. **(F)** Box plot showing the age distribution of Gly and Non-Gly PDA patients. *p*-value from Wilcoxon Rank-Sum test. **(G)** Kaplan Meier curve showing the cumulative survival probability of patients from the four glycolytic subtypes. ^*^p < 0.05; ^**^p < 0.01; ^***^p < 0.001.

To identify the candidate PDA glycolysis subtypes, we performed an integrative analysis of expression and CNVs of 38 glycolytic genes using RNA-Seq and whole genome sequencing data from 176 TCGA PDA samples. Clustering analysis of glycolytic genes expression data (Supplementary Table 1), resolved two main patient clusters corresponding to distinct PDA glycolytic subtypes (Figure 1A). On the basis of the gene expression differences between these clusters we named these two main PDA subtypes as Glycolytic (*Gly, n* = 58) and Non-Glycolytic (*Non-Gly, n* = 118). The Gly subtype was characterized by an over-expression of genes like *TPI1, GAPDH, ENO1, LDHA*, and *PGK1* (Figure 1B).

Basing on this clustering analysis, Gly tumors can be further subdivided into two subtypes defined as High Glycolytic (HG, *n* = 32) and Very High Glycolytic (VHG, *n* = 26). Similarly, the Non-Gly tumors can be subdivided into Low Glycolytic (LG, *n* = 104) and Very Low Glycolytic (VLG, *n* = 14) subtypes. VHG patients were characterized by the highest glycolytic genes expression while VLG patients were characterized by the lowest glycolytic genes expression (Supplementary Table 1).

Since an extensive stromal component is a hallmark of PDA (4), we predicted the stromal and the immune infiltrate of our set of PDA samples using *ESTIMATE* tool (28). Interestingly, the Non-Gly subtype was characterized by a higher predicted stromal/immunological component compared to the Gly subtype (*p* = 3.20 ·10^−4^) (Figure 1A). ESTIMATE analysis of the four subtypes confirmed that LG tumors were associated with highest stromal and immune infiltrate (p < 0.001), which decreases in HG, VHG, and VLG tumors (Supplementary Figure 1A).

To verify whether glycolytic genes expression levels were biased by the sample purity, we retrieved the *p*-value of the correlation between tumor purity and gene expression computed by TIMER tool (29). The data showed that the expression of only four out of 38 genes (*HK3, FBP1, PKLR, GCK*) was significantly affected by tumor purity (p < 0.01) but none of them was significantly differentially expressed between Gly and Non-Gly subtypes (Supplementary Table 1).

The number of CNVs was also significantly different between the Gly and the Non-Gly subtypes with Gly tumors characterized by an higher number of both CNV gain amplification (*p* = 3.20 ·10^−4^) and loss/deletion (*p* = 1.63 ·10^−10^) (Figure 1A). Interestingly, we observed a significant correlation between the expression level and the CNV status of *GAPDH, ADPGK, TPI1, PKM, PGK1, ENO1, GPI*, and *PGAM5* (adj. *p* < 0.001) (Supplementary Figure 1B).

The analysis of *CDKN2A, KRAS, MYC, SMAD4, TP53* genes, whose alterations are known to contribute to PDA development (34, 35) showed a significantly higher expression of *CDKN2A* and *KRAS* and a lower expression of *SMAD4* and *TP53* (Figure 1C). CNV events in *CDKN2A, KRAS, MYC, SMAD4*, and *TP53* genes were also significantly different between the two subtypes (Figure 1D). Furthermore, *KRAS* and *TP53* are slightly more mutated in the Gly subtype (Supplementary Figure 1C). CNVs and expression levels of these genes were also analyzed in the four subtypes confirming the differences in expression levels and CNV of glycolytic and non-glycolytic tumors (Supplementary Figures 1D,E).

To test the hypothesis that different glycolytic PDA subtypes correspond to patients with different clinical characteristics and tumor aggressiveness, we performed statistical analysis on patients clinical features (Supplementary Table 2A). Coherently with the loss of *TP53* expression, the Gly subtype was characterized by a significantly higher mutational counts compared to the Non-Gly subtype (Figure 1E and Supplementary Table 2B). Noteworthy, patients with Gly subtype develop tumor at lower age (Figure 1F) and the tumor size at resection is significantly larger than the non-glycolytic tumors (Supplementary Figure 1F). These patients are also characterized by a worse prognosis that leads to a significantly higher fraction of patients deceased or with recurred/progressed disease (Supplementary Figures 1G,H).

The analysis of clinical features of patients belonging to the four subtypes revealed that VHG tumors are slightly more mutated compared to the HG subtype (*p* = 0.048) (Supplementary Table 2B). VLG and VHG were significantly different in terms of months of patient survival and overall survival status (*p* = 0.011), months of disease freedom (*p* = 0.033), and initial tumor sample weight (*p* = 0.012). Coherently with these results, patients with Gly tumors were characterized by a lower survival (median = 17.02 months) time compared with Non-Gly PDA patients (median = 22.70 months) (*p* = 0.019) (Supplementary Figure 1I). Among the four subtypes identified, LG patients were characterized by higher survival time (median = 20.6 month) compared to the other subtypes (from 15.11 to 17.9 months) (*p* = 0.009) (Figure 1G and Supplementary Figure 1J). We investigated the impact of clinical features on survival time using multivariate Cox regression analysis (Supplementary Table 2C). From this analysis we observed that the lymph node positive status (N1) is a negative factor for survival (HR = 1.86, *p* = 0.023) and we confirmed that belonging to the Non-Glycolytic subtype is a positive factor for survival (HR = 0.19, *p* = 0.018).

To verify that the observed differences among PDA samples were not specific of the TCGA dataset, we performed an analysis of RNA-Seq data from an independent cohort of 99 PDA samples from ICGC. The analysis confirmed the presence of distinct glycolytic subtypes (Gly *n* = 29, Non-Gly *n* = 62) (Supplementary Figure 1K) as observed for the TCGA cohort, but also revealed significant differences in terms of survival between the two groups (*p* = 0.034) (Supplementary Figure 1L).

Taken together, these results suggest that Gly subtype is more aggressive than Non-Gly subtype due to an extended transcriptomic alteration of key glycolytic genes.

### 3.2. Immuno-Transcriptomic Analysis Revealed That Glycolytic PDA Subtypes Are Depleted in Infiltrating CD4+ T Cells

**Figure 2:**
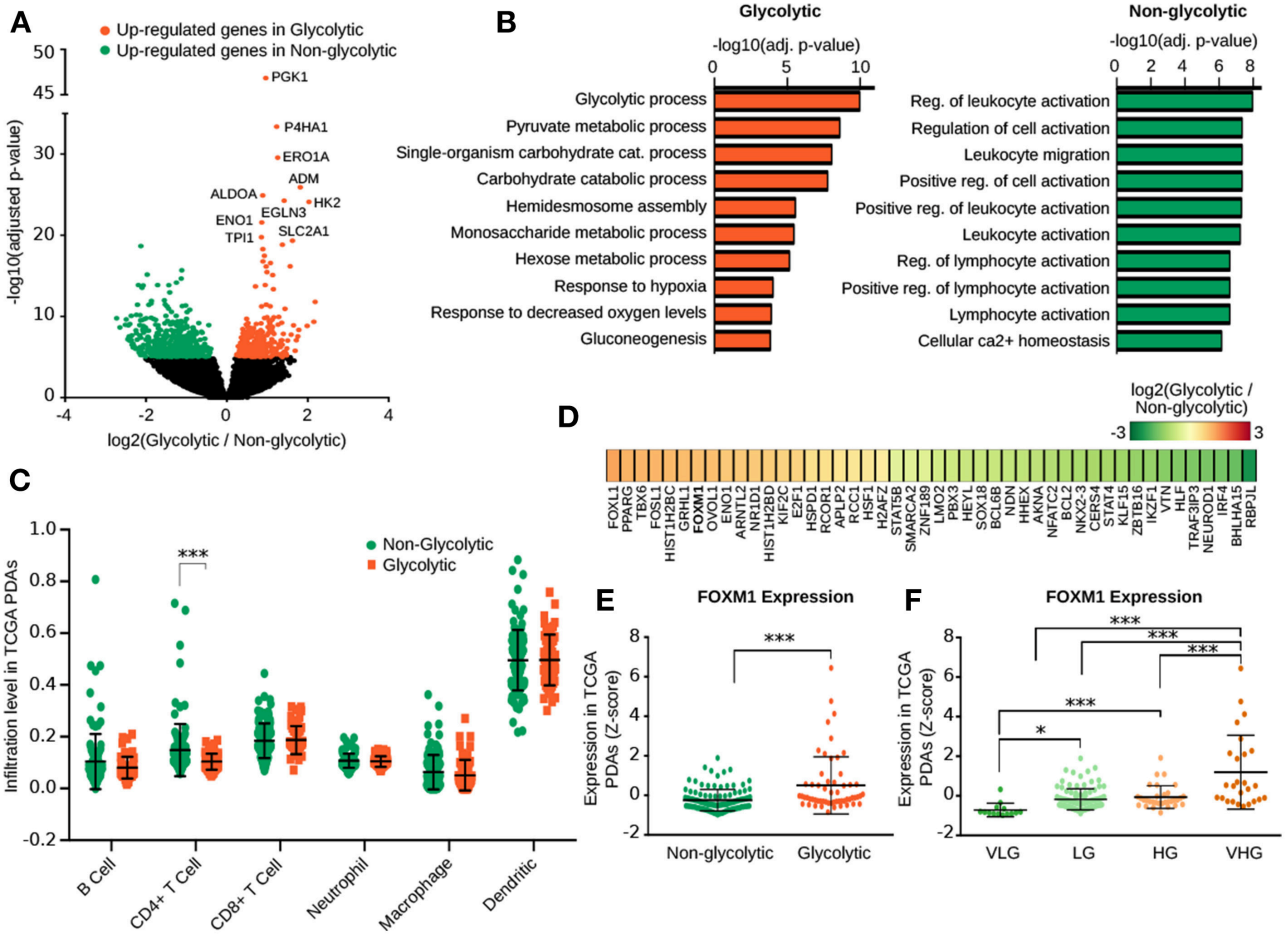
Transcriptional and immunological differences among PDA metabolic subtypes. **(A)** Volcano plot shows the expression fold change and the adjusted *p*-value of genes Differentially Expressed (DE) between the Gly and Non-Gly PDA subtypes. **(B)** Bar plot reporting the adjusted *p*-value of Gene Ontology biological processes enriched in genes up-regulated in the Gly (top) and Non-Gly (bottom) PDA subtype. **(C)** Dot plot reporting the levels of infiltrating immune cells computed with TIMER in the Gly and Non-Gly tumor subtypes. **(D)** Heat map reporting the log2FC of expression of transcription factors DE between the Gly and Non-Gly PDA subtypes. *FOXM1* (highlighted in bold) was also predicted as upstream regulators by the Enrichr analysis. **(E)** Dot plot reporting the level of *FOXM1* expression in the Gly and Non-Gly tumor subtypes. *p*-value by Wilcox Rank-Sum test. **(F)** Dot plot reporting the level of *FOXM1* expression in the four glycolytic PDA subtypes. *P*-value was computed using Wilcoxon Rank-Sum test. ***p < 0.001.

To identify the transcriptional differences among the glycolytic PDA subtypes, we performed a differential expression analysis of the tumors. Between the Non-Gly and the Gly subtypes, we identified 763 highly significant Differentially Expressed (DE) genes (Adj. *p* < 1E-05), 296 of which over-expressed in Gly subtype and 466 over-expressed in the Non-Gly tumors (Figure 2A and Supplementary Table 3). As expected, among the 20 most significant DE genes in the Gly subtype we observed many glycolytic genes, including *PGK1, ALDOA, HK2, ENO1, TPI1, PFKP*, and *GAPDH* (Figure 2A and Supplementary Table 3). Furthermore, the gene coding for the glucose transporter member (*SLC2A1*) and many genes not involved in the glycolytic pathway, like *P4HA1, ERO1A, ADM*, and *EGLN3* were up-regulated in the Gly subtype. DE analysis, performed between VHG and HG subtypes, revealed glycolytic genes such as *PFKFB4, FBP1, ENO1, LDHA, TPI1, GAPDH*, to be up-regulated in VHG (Supplementary Table 3).

Functional enrichment analysis confirmed that the glycolytic process was enriched by genes up-regulated in the Gly subtype, while the immune response-related terms were enriched by genes up-regulated in the Non-Gly subtype (Figure 2B and Supplementary Table 4A). This result is coherent with the higher ESTIMATE score characterizing the Non-Gly tumors and it supports the evidence of an increased stromal/immune infiltrate in these samples.

To further explore the immunological infiltrate estimated for our PDA samples, we analyzed results from TIMER (29) reporting the infiltration level of six immunological cell populations predicted on the same data. Noteworthy, Gly subtypes were characterized by less dispersed infiltrate for most of the populations with a significant depletion in CD4+ T cell infiltrate (*p* = 7.565e-06) (Figure 2C). Immunological infiltrate estimated with TIMER in the four subtypes confirmed that LG tumors were characterized by a general higher infiltrate, particularly of infiltrating CD4+ T cells (Supplementary Figure 2).

Finally, we compared the DE genes with gene sets annotated as healthy or pathological phenotypes. Result of the comparison was that almost all normal pancreatic tissue-related terms were enriched in the Non-Gly subtype up-regulated genes. Furthermore, these genes were enriched in genes down-regulated in PDA (Supplementary Tables 4B,C). Conversely, genes up-regulated in the Gly subtype were enriched in genes up-regulated in esophagus samples and in different cancer types including PDA.

### 3.3. Identification of Upstream Regulators Characterizing the PDA Glycolytic Subtypes

Subsequently, we performed an analysis of transcriptional regulatory drivers of the glycolytic PDA subtypes. Among DE genes between Gly and Non-Gly subtypes, 46 genes code for a TF, including Forkhead-box protein L1 (*FOXL1*) and Peroxisome Proliferator-Activated Receptor gamma (*PPARG*) which were the most up-regulated TFs in the Gly subtype (Figure 2D and Supplementary Table 5A). Conversely, Recombining Binding Protein suppressor of hairless-like protein (*RBPJL*) and Class A basic helix-loop-helix protein 15 (*BHLHA15*) were the most up-regulated TFs in the Non-Gly subtype.

Using public gene-sets of validated TF-promoter interactions we observed that, coherently with the enriched biological processes, Hypoxia-Induced Factor 1-alpha (HIF1α) was predicted as upstream regulator of genes up-regulated in Gly subtype together with SMAD proteins (SMAD2, SMAD3, SMAD4), and *FOXM1* (Supplementary Table 5B). Instead, genes up-regulated in the Non-Gly subtype were enriched in Polycomb protein SUZ12 and Transcription regulator protein BACH1 promoter binding.

Noteworthy, FoxM1 was also significantly up-regulated in the Gly subtype and its expression was significantly higher in VHG compared to HG PDAs (Figures 2E,F). Since FoxM1 was previously identified as regulator of glycolytic genes in pancreatic cancer (36), we further investigated the correlation between *FOXM1* and glycolytic genes expression. Many glycolytic genes were positively correlated with *FOXM1* expression (adjusted *p* < 0.001), including *GAPDH* (*r* = 0.72), *TPI1* (*r* = 0.71), *HK1* (*r* = 0.458), *ENO1* (*r* = 0.372), and *LDHA* (*r* = 0.332) (Supplementary Table 5C), further supporting *FOXM1* as a candidate up-stream regulator of glycolytic genes expression.

### 3.4. Frequent Gain of chr12p Band 1 Genomic Region in Glycolytic Tumors

**Figure 3:**
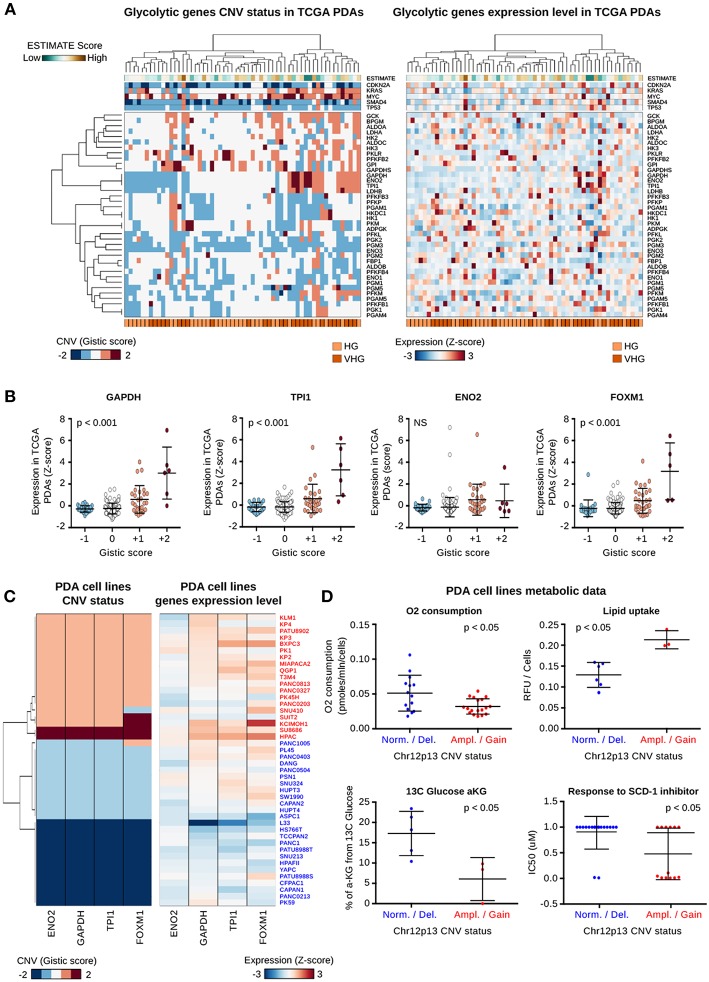
Characterization of chr12p13-amplified PDAs and cell lines. **(A)** Heat map showing the CNV status (right) and the normalized level of expression (left) of 39 genes coding for glycolytic enzymes in glycolytic patients. At top the expression and the CNV status of six genes involved in PDA tumorigenesis are reported. Green to brown colors indicate the ESTIMATE score proportional to the predicted stromal/immune component of each tumor sample. At bottom the two main glycolytic clusters are highlighted; HG, High Glycolytic; VHG, Very High Glycolytic. **(B)** Dot plots report the normalized expression level of four genes mapped at chr12p13 genomic region. The PDA datasets were subdivided on the basis of the CNV status of the same genomic region as reported by the Gistic score. *P*-value were computed using Pearson correlation analysis. **(C)** Heat map reporting the CNV status and expression level of the four genes mapped on chr12p13. Data were measured in PDA cell lines characterized by a gain/amplification (red) or a diploid/deletion (blue) of this region. **(D)** Dot plot reporting the level of metabolic and drug response features which are significantly different between the two groups of PDA cell lines characterized by the gain/amplification (Amp/Gain) or diploid/deletion (Dipl/Del) of chr12p13 genomic region.

As reported above, the PDA Gly subtype was characterized by bad prognosis, lower survival rate, lower stromal contamination, low CD4+ T cells infiltrate and high *FOXM1* expression. To further identify genomic drivers as recurrent genomic alterations of the glycolytic genes in Gly tumors, we performed a hierarchical clustering analysis of CNV data of HG and VHG PDAs (*n* = 58) (Figure 3A). The analysis highlighted a cluster of Gly tumors characterized by a co-gain/co-amplification of *TPI1, GAPDH, ENO2, LDHB* genomic regions and, it predominantly involved VHG tumors (*p* = 0.024).

Noteworthy, the co-gained/co-amplified genes are located at chr12 p arm band 1 (chr12p1) and in this genomic region are annotated also the *KRAS, GAPDH, ENO2*, and *TPI1, SLC2A3* (coding for the glucose transporter 3, GLUT3), the TP53-inducible glycolysis and apoptosis regulator (*TIGAR*) and the aforementioned *FOXM1* gene.

Interestingly, genes expression-CNV correlation analysis revealed that chr12p13 was the region harboring genes characterized by the highest positive correlation between CNV status and gene over-expression (*r* > 0.6) (Supplementary Tables 6A,B). *GAPDH, TPI1, FOXM1*, and *TIGAR*, all mapped on chr12p13, were associated with significant positive correlation between their CNV status and their expression, as shown in Figure 3B. Conversely, the expression of *LDHB* and *KRAS* which are located in chr12p band 1 sub-band 2 (chr12p12) did not correlate with their CNV status (Supplementary Figure 3A). This result suggests that chr12p13 is a specific regulatory genomic hub for the glycolytic pathway.

To verify that the co-gain/co-amplification was not specific of the PDA TCGA dataset, we performed the clustering analysis of CNV data on an independent cohort of 109 PDA patients (37) (Supplementary Figure 3B). The analysis confirmed that the chr12p13 genomic region is frequently associated with co-gain / co-amplification or co-deletion events involving *TPI1, GAPDH*, and *ENO2* genes.

Given the availability of public results from a metabolomic analysis of PDA cell lines (21), we investigated whether chr12p13 gain was related to metabolic changes.

To define two main cell line groups on the basis of their chr12p13 status, we analyzed gene expression and CNV data from 44 PDA cell lines from Cancer Cell Line Encyclopedia (CCLE) (27) (Supplementary Figure 3C and Supplementary Table 7A). Accordingly to the results observed in primary tumors and in PDA cell lines the gain/amplification positively correlated with *GAPDH, TPI1*, and *FOXM1* over-expression (Figure 3C). The analysis of the metabolomic data revealed that cell lines with a gain/amplification of chr12p13 were characterized by a lower oxygen consumption (*p* < 0.05), a higher lipid uptake (*p* < 0.05), and a lower incorporation of 13C glucose in α-ketoglutarate (*p* < 0.05) compared to diploid or chr12p13-deleted cell lines (Figure 3D). Incorporation of 13C glucose in malate and citrate was also higher in the Gly subtype albeit not statistically significant (*p* > 0.05) (Supplementary Figure 3D). A subset of ch12p13-amplified cell lines were also responsive to an inhibitor of Stearoyl-Coenzyme A Desaturase 1 (SCD-1) (p < 0.05, an enzyme involved in fatty acid elongation process.

These results suggest that transcriptionally active gain in chr12p13 leads to different metabolites consumption and production in pancreatic cancer cell lines.

### 3.5. TPI1 Is Abundant in Serum From Drug-Resistant PDA Patients

**Figure 4:**
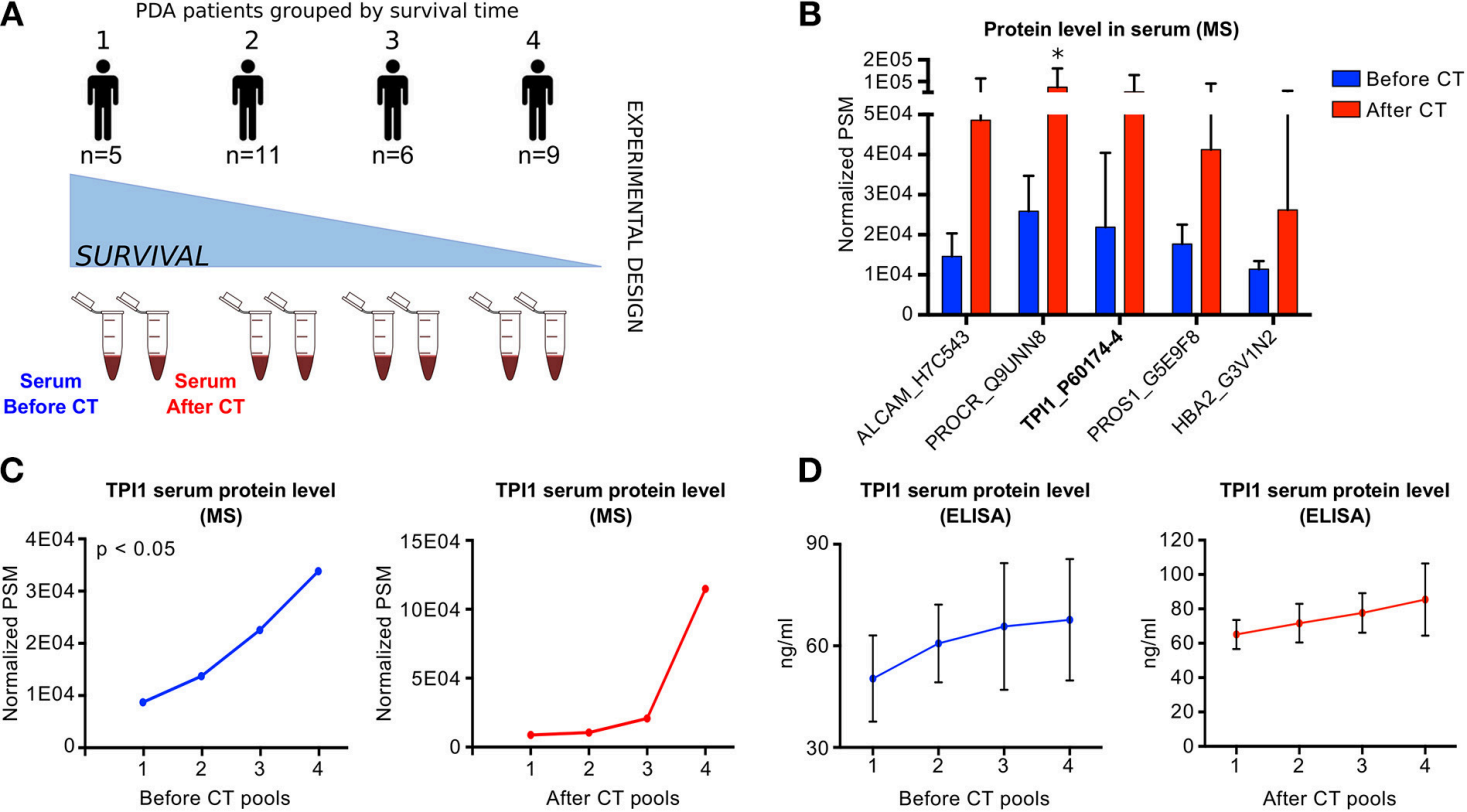
Proteomic analysis of PDA patients sera **(A)** Scheme of the experimental design of the free-circulating proteins proteomic analysis using pulled sera from PDA patients collected before and after chemotherapy (CT). Samples were divided in pools based on the months of disease-free survival. **(B)** Bar plot reporting the Peptide-Spectrum Matches (PSMs) of five circulating proteins associated with the highest log2 fold change between BCT and ACT samples. *P*-values were computed using Wilcoxon Rank-Sum test. *p < 0.05. **(C)** Line plot reporting the normalized TPI1 protein level measured in different pools of PDA patient sera collected before (left panel) or after CT (right panel). *P*-values were computed using Pearson correlation method. **(D)** Line plot reporting the normalized TPI1 protein level measured by ELISA assay applied on different pools of PDA patient sera collected before (left panel) or after CT (right panel).

To better delineate the role of the genes located on chr12p13 as clinical prognostic biomarkers, we conducted a pilot proteomic analysis of free circulating proteins in sera from a cohort of 31 PDA patients (Supplementary Table 8A). Since the majority of PDA patients are treated with gemcitabine-based CT, we also studied the effect of CT on circulating proteins codified by chr12p13 genes. The 31 patients were divided in four groups based on survival and response to CT (for detailed information see the Supplementary Methods section of the manuscript). In each group, sera were pooled and the pools protein content was analyzed using Q-TOF mass spectrometer (Figure 4A).

Mass Spectrometry (MS) data analysis revealed 886 protein isoforms detectable in sera, corresponding to 309 unique proteins. The most abundant circulating proteins were CD48 Before CT (BCT) and Glutathione Peroxidase 3 After CT (ACT), respectively. Cystatin C was abundant both BCT and ACT (Supplementary Table 8B).

By verifying the effect of CT on the proteins level, we observed a significant modulation of two proteins: the protein C receptor and the Dipeptidase 2 (Figure 4B and Supplementary Table 8C). Considering BCT and ACT proteins levels separately, respectively 73 and 28 proteins showed a significant trend across samples (Supplementary Table 8D).

Among the glycolytic enzymes, Triose Phosphate Isomerase (TPI1) was the unique glycolytic protein detectable in sera. Of note, TPI1 concentration increases after CT (Figure 4B) and, it was one of the most significant protein (BCT *p* = 0.0136), with a positive trend increasing from good to bad prognosis patients, both BCT and ACT (Figure 4C).

To validate MS results, we performed a TPI1 ELISA analysis on 23 sera belonging to the same patients cohort. The results shown in Figure 4D confirmed the positive trend of TPI concentration from good to bad prognosis patients in BCT and ACT.

These preliminary results suggest that sera TPI1 levels could be a marker of bad prognosis in advanced PDA patients.

## 4. Discussion

PDA is the result of a complex cross-talk between the tumor molecular profile and the metabolites produced by its microenvironment. Recently, many studies have identified PDA molecular subtypes using omic data. However, the identification of PDA metabolic subtypes is still missing. Metabolic classification could provide insights in the definition of metabolic targetable pathways and in the definition of new biomarkers.

Here, we presented a classification of PDAs based on the integration of the genomic and transcriptomic profiles of the glycolytic genes. Our classification groups tumors based on different aggressiveness, metabolic profiles and molecular characteristics. Our novel PDA classification suggests that might be a correlation between the chr12p13 gain and the increased levels of TPI1 in sera of patients with a bad prognosis.

In this paper, we clearly identified a glycolytic subtype of PDA characterized by a larger size at resection, higher rate of genomic alteration, an aberrant transcriptional profile with enrichment of FoxM1 expression, and a reduced predicted infiltration of CD4^+^T cells. Genomic analysis of this PDA subtype highlighted the gain of chr12p13 as a recurrent driver of glycolytic gene regulation. We observed the functional gain of chr12p13 in PDA cell line models and we identified a significant difference in the production of metabolites between cells with and without the functional gain. Clinically relevant, proteomic analysis of circulating proteins from PDA patients sera reveals that the level of TPI1 protein, coded from *TPI1* gene mapped at chr12p13, is increased in poor outcome patients before and after chemotherapy.

PDA can be classified in distinct molecular subtypes: the *exocrine-like/ADEX* subtype with expression of exocrine genes, the *quasi-mesenchymal/basal/squamous* subtype with mesenchymal gene expression associated with worse clinical outcome, and the *classical/luminal* subtype characterized by epithelial gene expression. Moreover, the *classical/luminal* subtype was recently grouped into Progenitor and Immunogenic subtypes based on the expression of early pancreas development and immune genes, respectively (17–19). By overlapping these PDA classifications with our glycolytic subtypes we confirmed that the glycolytic subtypes were enriched in the *quasi-mesenchymal/basal/squamous* tumors, while non-glycolytic subtypes were enriched in *exocrine-like/ADEX* and *classical* tumors (Supplementary Figure 3E and Supplementary Table 9).

It is worthwhile to note that the purity of PDA samples is an important aspect in PDA classification. Indeed, *immunogenic* or *ADEX* subtypes and the *exocrine-like* or *quasimesenchymal* subtypes could be erroneously derived considering the non-tumor cells gene expression in impure samples as recently demonstrated in Raphael et al. (34). In our study, glycolytic tumors were depleted in immune/stromal infiltrate while the non-glycolytic tumors are enriched in non neoplastic cells. This result was confirmed also by the analysis of a machine learning-based evaluation of the tumor infiltrates performed on TCGA histological data (30). The analysis of these data confirmed that VHG and HG tumors were characterized by a lower immune-infiltrate compared to the LG subtype (*p* < 0.05 and < 0.01, respectively) (Supplementary Figure 4A and Supplementary Table 11). Coherently with the higher immune-infiltrate, LG PDAs were characterized by a lower tumor cellularity compared to VHG and HG tumors (*p* < 0.01 and < 0.001, respectively) (Supplementary Figure 4B). However, the TIMER results show that the grade of purity have no effect on the genes expression in our samples with no statistically significant correlation between glycolytic genes expression and the tumor cellularity. Finally, the depleted immune/stromal infiltrate in the glycolytic subtype could be the result of the increased lactate production of these tumors. Indeed, high level of lactate can be secreted by cells and can induce an immunosuppressive microenvironment at the same time decreasing CD4+ T cells, as proved in Baek et al. (38), Hutcheson et al. (6), and Halbrook and Lyssiotis (7). Coherently with this hypothesis, we observed that the gene coding for the lactate/pyruvate transporter MCT-4 (*SLC16A3*) and *LDHA* coding for Lactate Dehydrogenase A enzyme are significantly up-regulated in the glycolytic subtype. These proteins are key promoters of the increment of lactate in the extracellular space (38, 39).

Metabolic and glycolytic enzymes, including Phosphoglycerate Kinase 1 (PGK1) Triose Phosphate Isomerase 1 (TPI1), Glucose-6-phosphate dehydrogenase (G6PD), Isocitrate dehydrogenase (IDHC), and Enolase 1 (ENO1) are highly expressed in PDA and they induce autoantibody and/or T cell responses in PDA (40–44). Furthermore, an aberrant glycolytic pathway plays a critical role in the modulation of tumor angiogenesis by altering drugs delivery and conferring a PDA phenotype which is resistant to CT (45, 46).

With the aim of identifying the molecular drivers of the glycolytic genes over-expression in PDA, we observed that chr12p13 gain clearly emerges as a recurrent alteration in VHG tumors and in PDA cell lines. This result is corroborated by results of Graham and colleagues (47) that included chr12p13 in their genomic CNV signature of glycolytic breast, ovarian, uterine, and lung tumors. The authors report that tumors associated with this signature is characterized by chromosomal instability and highly recurrent CNVs driving the metabolic adaptation to oxidative stress and the metabolic demand of highly proliferating cells. As already demonstrated by (48) the deletion of *SMAD4* tumor suppressor gene loci leads to a loss of neighboring genes in PDA (i.e., malic enzyme 2 *ME2*). In our study, we propose that the chr12p13 gain leads to the over-expression of genes annotated in this genomic region (i.e., *GAPDH, TPI1, FOXM1*) creating cancer-specific metabolic addiction.

Analysis of TFs expression level in glycolytic PDAs showed that chr12p13 regions harbor also the *FOXM1* gene, whose expression level positively correlates with the chr12p13 CNV status. *FOXM1* is highly expressed in aggressive tumors including PDA (49, 50) regulating tumor growth proliferation, migration, and angiogenesis (51). Moreover, *FOXM1* is a hypoxia-induced gene (52) and its transcriptional activity on *LDHA* and *PGK1* was reported in PDA cell line (53). We provided the evidence that in PDA primary tumors FOXM1 can be co-amplified with genes coding for critical glycolytic enzymes adding further insights on the role of this TF in the regulation of the PDA metabolism.

The chr12p13 signature is present both in patients and cell lines. By analysis of PDA cell lines metabolic data, we added further evidence on the function effect of chr12p13 gain on cell metabolism. The chr12p13-gained cells were characterized by a higher lipid uptake and sensitivity to inhibitor of SCD-1, an enzyme catalyzing the rate-limiting step of mono-unsaturated fatty acids synthesis. Analysis of metabolite produced by these cells showed a lower amount of lipids and a higher amount of spermine, glutamine, and glutathione disulfide (Supplementary Table 7B). We did not observed significant overlap between our classification and the one proposed in Daemen et al. (21) for PDA cell lines (chi-square *p* >0.05). However, metabolic data obtained from cell lines should be compared carefully with the one from primary tumors since the interaction with the tumor microenvironment is not completely taken into consideration in cell lines models. This is especially true in PDA, where the presence of stromal and immune cells extensively influences the tumor cell metabolism. Nevertheless, the above result show us that the chr12p13 signature is a genomic characteristic of primary tumor cells that excludes a contamination of stroma. We hypothesize that the over-expression of *GAPDH, TPI*, and *TIGAR* in chr12p13-gained cells leads to a metabolic flow deviation of glycolysis toward other metabolic pathways like the Pentose Phosphate Pathway (PPP), the fatty acid elongation, and the glutathione production. Of note, functional enrichment analysis of genes over-expressed in the Gly subtype highlighted that some genes belong to the PPP and the TriCarboxylic Acid (TCA) cycle. DE analysis of these genes revealed that six genes coding for PPP enzymes were significantly over-expressed in Gly tumors while four TCA cycle genes showed an altered expression in these tumors (Supplementary Table 10). These results suggest that the Gly tumors phenotypes relies most in an altered glycolysis and PPP while TCA cycle is only partially perturbed as compared to the Non-Gly subtype. However, a more extensive metabolomic analysis is needed to better understand the extension of the metabolic rewiring in these tumors.

Our proteomic analysis of sera from an independent cohort of pancreatic cancer patients showed TPI1 as one of the most abundant protein in low survival patients before and after CT. Our analysis shows that TPI1, involved in the identification of the aggressive PDA types, is associated with an increased level of its protein in the serum. Therefore, we suggest that TPI1 could be further investigated as a prognostic marker, since its level is gradually increased as prognosis worsens. The observed trends are not changed by CT treatment, that is only able to further increase the level of TPI1. The confirmation of this hypothesis will be provided by analyzing a paired genomic and sera proteomic data from a larger cohort of PDA patients before and after CT.

## 5. Conclusions

Emerging strategies to treat PDA include the characterization of the key complex molecular interactions that lead to carcinogenesis. These complex molecular and metabolic interactions may represent tumor vulnerabilities.

Our results identified four PDA subtypes with the presence of at least two cancer metabolic vulnerabilities linked to glycolytic pathway: one related to the dependency of specific transcriptional regulators like FoxM1, the other one related to the metabolic flow redirection toward fatty acid elongation instead of oxidative phosphorylation. Since many drugs targeting TFs or metabolic enzymes are emerging, our results suggest that an efficient combination of them can be used with common chemotherapy to properly treat PDA progression and the onset of drug-resistant disease.

## Data Availability

All relevant data are available within the Article and Supplementary Files, or available from the authors upon request.

## Ethics Statement

Biological samples were retrieved from 31 patients affected by PDA enrolled in ENOAPA project (https://www.epiclin.it/enoapa). All of them provided written informed consent for the research use of the biological samples, all the procedures were conducted in accordance with the Declaration of Helsinki and ethical approval for the PDA study was given by the Ethical Committee of the AOU Città della Salute e della Scienza di Torino, Turin, Italy.

## Author Contributions

LF, GF, MB, and FC designed the project. LF and GF equally contributed to perform computational analyses and to wrote the manuscript. GM designed and performed the proteomic and ELISA analyses. HK, WH, and AM support proteomic analyses. DG, RS, and MS contributed to serum samples collection. AE performed statistical analysis of patients analyzed using MS. MB and FC supervised bioinformatic analyses. MC and SH allowed and supervised proteomic data analysis. FN and FC equally supervised the overall work. All authors have read and approved the manuscript.

### Conflict of Interest Statement

The authors declare that the research was conducted in the absence of any commercial or financial relationships that could be construed as a potential conflict of interest.
