# Supplementary material for: Integrative Analysis of Novel Metabolic Subtypes in Pancreatic Cancer Fosters New Prognostic Biomarkers
Source: Front Oncol. 2019 Feb 27;9:115. doi: 10.3389/fonc.2019.00115 (PMC6400843; doi:10.3389/fonc.2019.00115)
Supplement: Supplementary file 12 [file Data_Sheet_1.PDF]

Supplementary Material: Integrative analysis of novel metabolic subtypes in pancreatic cancer fosters new prognostic biomarkers

SUPPLEMENTARY METHODS

Selection of glycolytic genes

The list of genes coding for glycolytic enzymes was defined using Gene Ontology (GO) by selecting the GO Term "*Glycolytic process*" (GO:0006096). The annotations from Ensembl (release 86) of 71 genes annotated to this ontological term were isolated using BioMart tool Kinsella et al. (2011). Among the genes coding for glycolytic enzymes, a subset of 38 genes, strictly belonging to the glycolytic pathway, was selected. Since our study was not focused on glycolysis in sex-specific tissues the genes expressed in testis tissue (*GAPDHS, PGK2*) were excluded from the analysis. Conversely, the *LDHB* gene coding for isoform H of LDH involved in the catabolism of lactate was included in our gene list.

PDA DNA and RNA sequencing datasets

Processed RNA-sequencing (RNA-Seq) and Whole Genome Sequencing (WGS) data of 176 Pancreatic Ductal Adenocarcinoma (PDA) patients were retrieved from The Cancer Genome Atlas (TCGA) consortium. Specifically RNA-Seq data of TCGA-PAAD project were downloaded from the Genomic Data Common portal (GDC) Grossman et al. (2016) by considering the gene expression levels normalized as Fragment Per Kilobase Mapped reads (FPKM). For each sample, the paired processed Copy Number Variants (CNVs) and mutational data were obtained from UCSC Xena (`http://xena.ucsc.edu`). CNV data were retrieved as GISTIC-thresholded values in which genes CNV level are discretized in five numerical values: 2, genomic amplification; 1, genomic gain; 0, Diploid status; -1, Heterozygous deletion; -2, Homozygous deletion Mermel et al. (2011). Gene-level non-silent mutations data were obtained from the *broad automated* dataset provided by Xena. TCGA PDA patient clinical data were obtained from cBioPortal for Cancer Genomics Gao et al. (2013)(study name: *Pancreatic Adenocarcinoma, TCGA Provisional*).
RNA-Seq expression data of 99 patients belonging to the PACA-AU cohort from International Cancer Genome Consortium (ICGC) were retrieved from (`http://docs.icgc.org` Scarlett et al. (2011)). The gene expression levels were normalized as FPKM . The associated clinical data were also retrieved.
GISTIC-thresholded CNVs tumor data of a cohort of 109 PDA patients from the University of Texas Southwestern (UTSW) Witkiewicz et al. (2015) were retrieved from CbioPortal (study name: *Pancreatic Cancer (UTSW, Nat Commun 2015)*) as well as GISTIC-thresholded CNV and Z-score-normalized expression data of 44 PDA cell lines from the Cancer Cell Line Encyclopedia (CCLE) Barretina et al. (2012) (study name: *Cancer Cell Line Encyclopedia (Novartis/Broad, Nature 2012)*).

Definition of PDA glycolytic subtype

PDA glycolytic subtypes were defined by hierarchical clustering analysis applied on Z-score-transformed TCGA RNA-Seq expression data. The hierarchical clustering analysis was performed using *Heatmap.2* R package using the Euclidean distance and the *Ward.D2* clustering method.

The clustering defined two main PDA clusters composed of PDA defined as Glycolytic (Gly) and Non-Glycolytic (Non-Gly) subtype. The clustering results were used also to separate the Gly subtype in two patient groups, we named them as High Glycolytic (HG) and Very High Glycolytic (VHG). The Non-Gly subtype was also subdivided into two subtypes named Low Glycolytic (LG) and Very Low Glycolytic (VLG). Hierarchical clustering analysis was applied on ICGC (Expression data), UTSW (CNV data), CCLE (CNV data) datasets.

Analysis of the genomic and transcriptomic differences in glycolytic genes among the glycolytic PDA subtypes

Differential expression analysis between Z-score-normalized RNA-Seq expression level was performed using Wilcox Rank Sum test and p-value were corrected using the Benjamini-Hochberg (BH) method.
Differential CNV status and mutational analyses between glycolytic subtypes were performed using the Chi-square test and p-value were corrected using the BH method.
Expression-CNVs correlation analysis was performed using Pearson method. Adjusted p-values have been graphically represented as bar plots.

Clinical data analysis and survival analysis

Statistical analysis on PDA clinical data of TCGA and ICGC cohorts was performed using both R and Graph Pad Prism. For both cohorts only covariates measured for at least half of patients were considered. P-values has been calculated using Wilcoxon Rank-Sum test for continuous data and Chi-square test for categorical parameters. The analysis was performed between Gly and Non-Gly subtypes and between VHG and HG groups.
Estimates of the cumulative survival distributions were computed by the Kaplan-Meier method, and the differences between groups were compared using the log-rank test.
The significance of each clinical parameter data was also evaluated using multivariate Cox proportional hazard regression model implemented in *survival* R package with default parameters. Only covariates with at most one NA value were considered.
The significance of clinical data was also evaluated using multivariate Cox proportional hazard regression model using the *Coxph* function of Survival R package

Results were computed using and were graphically represented using Graph Pad Prism 6.

Evaluation of the immunological and stromal infiltrate

The amount of the immunological and stromal infiltrate among PDA subtypes in TCGA study was evaluated using ESTIMATE Yoshihara et al. (2013) by downloading from `http://bioinformatics.mdanderson.org/estimate/`, the Stromal and Immunological Scores pre-computed for TCGA-PAAD cohort based on RNA-Seq v2 data. The population of tumor-infiltrating immune cells were inferred using TIMER Li et al. (2016) data at `https://cistrome.shinyapps.io/timer`.
Data of the TCGA Pancreatic Adenocarcinoma (PAAD) study were downloaded from the Estimation module. Statistical differences among the four subtypes were computed using Wilcoxon Rank-Sum test.
TIMER data were used also to evaluate the influence of sample purity on glycolytic genes expression by retrieving the purity-corrected partial Spearman correlation and the statistical significance from the Gene module.
The level of immune infiltrate of the TCGA PDAs was evaluated by analyzing the data from Saltz

et al. (2018). Specifically, considering the data associated with the publication, the percentage of tumor-infiltrating lymphocytes (”til_percentage” parameter) was retrieved for 160 TCGA PDAs out of the 176 samples analyzed in this study.

Differential genes expression analysis

Differentially Expressed (DE) genes among the Gly and Non-Gly and VHG and HG subtypes were identified using *DESeq2* R package Love et al. (2014). Only genes associated with an adjusted p-value < 1E-05 were considered as significantly DE. DE analysis of genes annotated to the PPP or the TCA cycle was performed by considering the gene product of the GO term *pentose-phosphate shunt (GO:0006098)* and ”tricarboxylic acid cycle (GO:0006099)”, respectively.

Gene sets enrichment and Transcription Factors (TF) analysis

Gene set analysis was performed using Enrichr Kuleshov et al. (2016). Molecular pathways enriched in the list of DE genes were identified by considering the gene set libraries *Gene Ontology (GO) Biological Processes*, *KEGG*, *Wiki Pathways*, *Reactome*, *NCI-Nature*, and *Panther*. *Jansen Tissue*, *Human Gene Atlas*, *GTEx Up*, and *GTEx Down* gene set libraries were used to compute the enrichment of DE genes in gene set related to normal tissues. Conversely, *OMIM Disease*, *Disease_Perturbations_from_GEO_up*, *Disease_Perturbations_from_GEO_down*, and *Jensen Disease* libraries were used to compute the enrichment in disease-related gene sets. Only the top 20 terms associated to adjusted p-value < 0.001 were considered. Gene Set Enrichment Analysis (GSEA, Subramanian et al., 2015) was performed using the list of DE genes sorted by log2FC. The *preRanked* mode of the program was applied and only gene sets associated to FWER < 0.05 were considered. The prediction of TFs regulating DE gene expression was performed using Enrichr by considering the *ChEA* and *ENCODE_and_ChEA_Consensus_TFs_from_ChIP-X* gene set libraries reporting gene set annotated to validated TF-promoter binding. Only the top 20 terms associated to adjusted p-value < 0.001 were considered. Identification of TFs among DE genes was performed using the annotations from AnimalTFDB Zhang et al. (2014).
Identification of genes correlated in expression with *FOXM1* was performed using Pearson method. Only genes associated with BH adjusted p-value < 0.001 were considered.

Metabolomic data analysis

Metabolomics data of pancreatic cancer cell lines were retrieved from supplementary material of Daemen et al. (2015). Cell lines used in this study were classified as Gained/Amplified or Diploid/Deleted basing on their CNV status of *TPI1*, *GAPDH*, *ENO2* and *FOXM1*. The CNV status were retrieved from the CCLE data. Analysis of metabolic differences among cell line groups was performed by Wilcoxon Rank-Sum test. Analysis of metabolite abundances was performed by considering data from Broad Profiling and Energy platforms from Daemen et al. (2015). For each metabolite, the average abundance across replicates was computed and the difference between cell line groups analyzed by Wilcoxon Rank-Sum test.

ELISA

ELISA was performed with the commercial kit AssayMax Human TIM ELISA Kit (AssayPro, St. Charles, MO) following manufacturer instructions. Twenty-three patients sera, before and after CT, were tested individually.

Mass Spectrometry (MS)-based proteomic analyses

From 2006 to 2012, 37 patients with PDA were enrolled in this study and provided serum samples. All patients were not subjected to surgery and were treated with gemcitabine-based CT (gemcitabine with oxaliplatin or alone). Serum samples were isolated from venous blood before CT and at each observation after cycles of CT and stored at -80 °C until use.

Based on their survival and response to CT, patients were subdivided in 4 groups. Sera of patients within each group were pooled and 300 μl of serum from each pool were used for MS analysis. Depletion of IgG-, IgM-, IgA-bound proteins was performed using HiTrap Protein G HP 1 ml (GE HealthCare Life Sciences), HiTrap IgM purification HP 1 ml (GE HealthCare Life Sciences) and anti-human IgA (chain specific)-agarose antibody (Sigma). Depletion HLA-I and -II complexes was performed using the binding of anti-HLA-I or anti-HLA-II to Glycolink micro immobilization kit (Life Technologies). Finally, free circulating proteins were collected.

Each sample was subjected to immuno-depletion (Hu-14 column, $10xmm$; Agilent Technologies, Wilmingyon, DE). After reduction with TCEP (tris(2-carboxyethyl)phosphine; Thermo), sample were tagged with iodoacetyl tandem mass tag reagents (Thermo) and 2D-HPLC fractionation and trypsin digestion were performed as previously described Wang and Hanash (2011). MS analysis was performed by Q-TOF micro (Micromass, Manchester, United Kingdom).

nanoAcquity UPLC system coupled on-line with SYNAPT G2-S mass spectrometer (Waters, Milford, MA) was used for the separation of pooled digested protein fractions.The system was equipped with a Waters Symmetry C18 nanoAcquity trap-column (180 μm x 20mm) and a C18 analytical column with built-in emitter (75 μm x 250 mm, Column Technology, Inc).

The temperature of the tray compartment in auto-sampler was 6 °C. Approximately 1μg of protein digest was loaded onto the trap-column through a 20μL sample-loop using 98% mobile phase A (0.1% formic acid in 2% ACN) at a flow rate of 8μL/min for 5 min.The desalted peptides eluted from the analytical column at a flow rate of 500nl/min with a gradient elution included an increase from 3% to 25% mobile phase B (0.1% formic acid in 98% ACN) over 100 min, 25% to 85% mobile phase B for 7 min, a wash step to hold at 85% mobile phase B for 3 min, and a re-equilibration step at 3% mobile phase B for 10 min. The lock mass, 300 fmol/μL of [Glu1] fibrinopeptide solution prepared with 0.1% formic acid in 30% ACN, was delivered from the auxiliary pump of the nanoAcquity UPLC at a flow rate of 0.2μL/min to the reference sprayer of the NanoLockSpray source.

LC-HDMSE Data was acquired in resolution mode with SYNAPT G2-S using Waters Masslynx (version 4.1, SCN 851). The capillary voltage was set to 2.80 kV, sampling cone voltage to 30 V, source offset to 30 V, and source temperature to 100°C.Mobility utilized high-purity N2 as the drift gas in the IMS TriWave cell. Pressures in the helium cell, Trap cell, IMS TriWave cell, and Transfer cell were 4.50 mbar, 2.47e-2 mbar, 2.90 mbar, and 2.53e-3 mbar, respectively. IMS wave velocity was 600 m/s, helium cell DC was 50 V, Trap DC bias was 45 V, IMS TriWave DC bias was 3 V, and IMS wave delay was 450μs.The mass spectrometer was operated in V-mode with a typical resolving power of at least 21,000. All analyses were performed using positive mode ESI using a NanoLockSpray source. The lock mass channel was sampled every 60 s. The mass spectrometer was calibrated with a [Glu1] fibrinopeptide solution (300 fmol/μL) delivered through the reference sprayer of the NanoLockSpray source. Accurate mass LC-HDMSE data was collected in an alternating, low energy (MS) and high energy (MSE) mode of acquisition with mass scan range from m/z 50 to 1800. The spectral acquisition time in each mode was 1.0 s with a 0.1-s inter-scan delay. In low energy HDMS mode, data was collected at constant collision energy of 2 eV in both Trap cell and Transfer cell. In high energy HDMSE mode, the collision energy was ramped from 25 to 55 eV in the Transfer cell only. The RF applied to the quadrupole mass analyzer was adjusted such that ions from m/z

300 to 2000 were efficiently transmitted, ensuring that any ions observed in the LC-HDMSE data less than m/z 300 were known to arise from dissociations in the Transfer collision cell.

The continuum LC-HDMSE data was processed using ProteinLynx Global Server (PLGS, version 3.0.1, Waters, Milford, MA). The low-energy (HDMS) and high-energy (HDMSE) data were background subtracted, de-isotoped and charge-state reduced to the corresponding monoisotopic peaks. Each monoisotopic peak was then lock-mass corrected to yield the accurate mass measurement. The lock mass for charge 2 was 785.8426 Da with a lock mass windows of 0.25 Da. Fragment ions and their corresponding precursor ions were aligned together based on the profile of mobility drift time as well as the chromatographic retention time. A low energy threshold of 250 counts, and a high energy threshold of 50 counts, and an intensity threshold of 1250 counts were chosen for generating spectra for protein identification and quantification.

Protein was identified by searching the processed spectra against the complete proteome set of H. sapiens from Uniprot. A fixed iodoTMT modification for Cysteine, a variable oxidation modification for Methionine were specified. One trypsin miscleavage was allowed, and the default settings in PLGS for the precursor ion and fragment ion mass tolerance were used. The search thresholds used were: minimum fragment ion matches per peptide, 3; minimum fragment ion matches per protein, 7; minimum peptides per protein, 1; and false positive value, 4.

The 6-plex TMT labeled peptides were then normalized using the quantile normalization method while comparison of protein abundance before and after CT using DESeq2 R package. Analysis of protein abundances trends among the 4 sample pools was performed using Pearson Correlation test.

Classification of TCGA PDA glycolytic subtypes using public PDA molecular subtypes

The TCGA PDA samples used in our analysis were classified into different PDA molecular subtypes based on PDA classification proposed in three different studies Collisson et al. (2011); Moffitt et al. (2015); Bailey et al. (2016). To avoid influences of sample cellularity in the re-classification, only the samples and associated classification reported in Raphael et al. (2017) were considered. Compared to our set of TCGA PDA samples, the list of samples used in Raphael et al. (2017) does not includes 18 PDAs classified as VHG (n=3), HG (n=1), LG (n=6), VLG (n=8) in our analysis.

REFERENCES

Kinsella RJ, Kähäri A, Haider S, Zamora J, Proctor G, Spudich G, et al. Ensembl biomarts: a hub for data retrieval across taxonomic space. *Database* **2011** (2011).

Grossman RL, Heath AP, Ferretti V, Varmus HE, Lowy DR, Kibbe WA, et al. Toward a shared vision for cancer genomic data. *New England Journal of Medicine* **375** (2016) 1109–1112.

Mermel CH, Schumacher SE, Hill B, Meyerson ML, Beroukhim R, Getz G. Gistic2. 0 facilitates sensitive and confident localization of the targets of focal somatic copy-number alteration in human cancers. *Genome biology* **12** (2011) R41.

Gao J, Aksoy BA, Dogrusoz U, Dresdner G, Gross B, Sumer SO, et al. Integrative analysis of complex cancer genomics and clinical profiles using the cbioportal. *Science signaling* **6** (2013) pl1.

Scarlett CJ, Salisbury EL, Biankin AV, Kench J. Precursor lesions in pancreatic cancer: morphological and molecular pathology. *Pathology* **43** (2011) 183–200.

Witkiewicz AK, McMillan EA, Balaji U, Baek G, Lin WC, Mansour J, et al. Whole-exome sequencing of pancreatic cancer defines genetic diversity and therapeutic targets. *Nature communications* **6** (2015).

Barretina J, Caponigro G, Stransky N, Venkatesan K, Margolin AA, Kim S, et al. The cancer cell line encyclopedia enables predictive modelling of anticancer drug sensitivity. *Nature* **483** (2012) 603–607.

Yoshihara K, Shahmoradgoli M, Martínez E, Vegesna R, Kim H, Torres-Garcia W, et al. Inferring tumour purity and stromal and immune cell admixture from expression data. *Nature communications* **4** (2013).

Li B, Severson E, Pignon JC, Zhao H, Li T, Novak J, et al. Comprehensive analyses of tumor immunity: implications for cancer immunotherapy. *Genome biology* **17** (2016) 174.

Saltz J, Gupta R, Hou L, Kurc T, Singh P, Nguyen V, et al. Spatial organization and molecular correlation of tumor-infiltrating lymphocytes using deep learning on pathology images. *Cell reports* **23** (2018) 181.

Love MI, Huber W, Anders S. Moderated estimation of fold change and dispersion for rna-seq data with deseq2. *Genome biology* **15** (2014) 550.

Kuleshov MV, Jones MR, Rouillard AD, Fernandez NF, Duan Q, Wang Z, et al. Enrichr: a comprehensive gene set enrichment analysis web server 2016 update. *Nucleic acids research* **44** (2016) W90–W97.

Zhang HM, Liu T, Liu CJ, Song S, Zhang X, Liu W, et al. Animaltfdb 2.0: a resource for expression, prediction and functional study of animal transcription factors. *Nucleic acids research* **43** (2014) D76–D81.

Daemen A, Peterson D, Sahu N, McCord R, Du X, Liu B, et al. Metabolite profiling stratifies pancreatic ductal adenocarcinomas into subtypes with distinct sensitivities to metabolic inhibitors. *Proceedings of the National Academy of Sciences* **112** (2015) E4410–E4417.

Wang H, Hanash S. Intact-protein analysis system for discovery of serum-based disease biomarkers. *Serum/Plasma Proteomics* (Springer) (2011), 69–85.

Collisson EA, Sadanandam A, Olson P, Gibb WJ, Truitt M, Gu S, et al. Subtypes of pancreatic ductal adenocarcinoma and their differing responses to therapy. *Nature medicine* **17** (2011) 500–503.

Moffitt RA, Marayati R, Flate EL, Volmar KE, Loeza SGH, Hoadley KA, et al. Virtual microdissection identifies distinct tumor-and stroma-specific subtypes of pancreatic ductal adenocarcinoma. *Nature genetics* **47** (2015) 1168.

Bailey P, Chang DK, Nones K, Johns AL, Patch AM, Gingras MC, et al. Genomic analyses identify molecular subtypes of pancreatic cancer. *Nature* (2016).

Raphael BJ, Hruban RH, Aguirre AJ, Moffitt RA, Yeh JJ, Stewart C, et al. Integrated genomic characterization of pancreatic ductal adenocarcinoma. *Cancer Cell* **32** (2017) 185–203.

SUPPLEMENTARY FIGURES

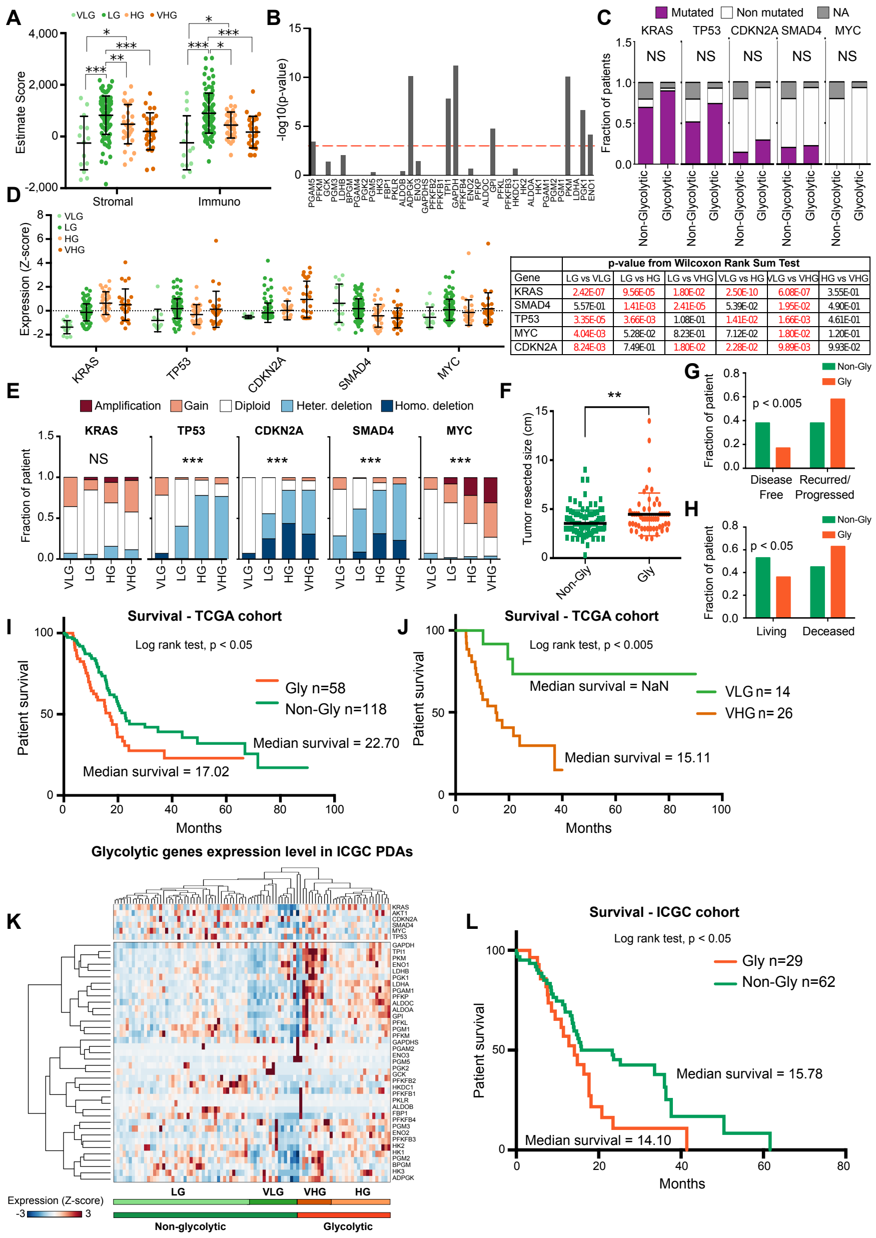

	p-value from Wilcoxon Rank Sum Test					
Gene	LG vs VLG	LG vs HG	LG vs VHG	VLG vs HG	VLG vs VHG	HG vs VHG
KRAS	2.42E-07	9.56E-05	1.80E-02	2.50E-10	6.08E-07	3.55E-01
SMAD4	5.57E-01	1.41E-03	2.41E-05	5.39E-02	1.95E-02	4.90E-01
TP53	3.35E-05	3.66E-03	1.08E-01	1.41E-02	1.66E-03	4.61E-01
MYC	4.04E-03	5.28E-02	8.23E-01	7.12E-02	1.80E-02	1.20E-01
CDKN2A	8.24E-03	7.49E-01	1.80E-02	2.28E-02	9.89E-03	9.93E-02

Figure S1. A. Dot plot illustrating the stromal and immunological score computed by ESTIMATE for the four glycolytic PDA subtypes. * = p-value < 0.05, ** = p-value <0.01, *** = p-value <0.001. **B.** Bar plot reporting the adjusted p-value of the correlations between expression level and CNV status of the 38 glycolytic genes. **C.** Bar plot illustrating the mutational status of five oncogenes/oncosuppressor in Gly and Non-Gly PDA subtypes. **D.** Dot plot reporting, for the four PDA glycolytic subtypes, the normalized expression level of five genes involved in PDA tumorigenesis in the four glycolytic subtypes. P-value from Wilcoxon Rank-Sum test. **E.** Bar plot showing, for the four PDA glycolytic subtypes, the distribution of CNV events of five genes involved in PDA tumorigenesis in the four glycolytic subtypes. P-values from Chi-square test ***= p-value <0.001. **F.** Dot plot reporting the size of resected tumors from Gly and Non-Gly PDA patients. P-value from Wilcoxon Rank-Sum test. ** = p-value < 0.01. **G-H** Histogram reporting the fraction of Gly and Non-Gly PDA among patients disease-free or with recurred/progressed disease (**G**) or among living or deceased patients (**H**). P-value from Chi-square test. **I.** Kaplan-Meier curve illustrating the cumulative survival probability of patients from the Gly and Non-Gly subtypes. **J.** Kaplan-Meier curve illustrating the cumulative survival probability of patients from the VLG and the VHG subtypes. P-values from log rank test. **K.** Heat map showing the normalized level of expression of 38 genes coding for glycolytic enzymes in 91 PDA samples from ICGC. At top the expression of five genes involved in PDA tumorigenesis is reported. **L.** Kaplan Meier curve illustrating the cumulative survival probability of ICGC patients from the Gly and the Non-Gly subtypes. P-value from log rank test.

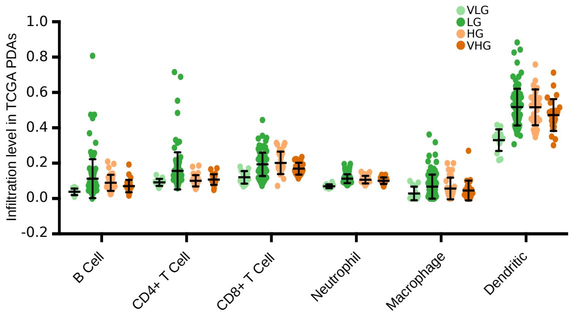

	p-value from Wilcoxon Rank Sum Test					
Population	LG vs VLG	LG vs HG	LG vs VHG	VLG vs HG	VLG vs VHG	HG vs VHG
B cell	1.10E-06	6.21E-01	7.88E-03	1.16E-05	4.51E-04	6.59E-02
CD4+ T cell	1.18E-05	2.33E-06	4.54E-04	3.88E-01	7.15E-02	2.94E-01
CD8+ T cell	3.11E-05	3.68E-01	1.30E-01	3.55E-05	4.51E-04	4.56E-02
Neutrophil	1.01E-08	2.73E-01	4.02E-02	7.60E-09	7.65E-07	3.55E-01
Macrophage	1.51E-02	3.17E-01	7.74E-02	9.02E-02	1.92E-01	6.16E-01
Dendritic	1.64E-08	8.27E-01	3.54E-02	1.01E-08	2.76E-06	1.32E-01

Figure S2. Dot plot reporting the levels of infiltrating immune cells computed with TIMER for the four PDA glycolytic subtypes. P-value from Wilcoxon Rank-Sum test.

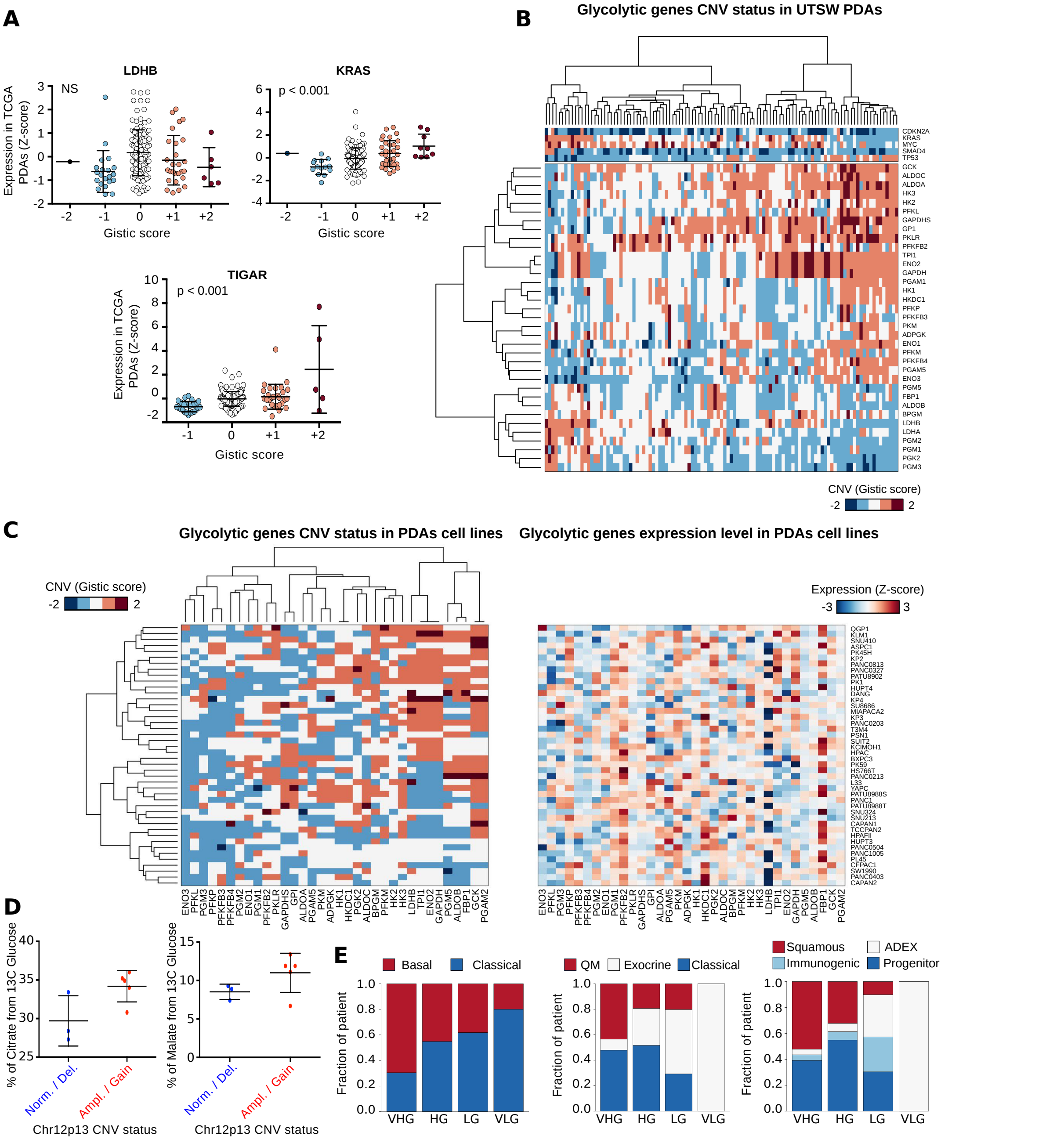

Figure S3. **A.** Heat map showing the Copy Number Variation (CNV) status of 38 genes coding for glycolytic enzymes in 109 PDA samples from University of Texas Southwestern (UTSW). At top is reported the CNV status of five genes involved in PDA carcinogenesis. **B.** Dot plot reporting normalized expression levels of three genes mapped on chr12p13 genomic region. The PDA datasets were subdivided based on the CNV status of the same genomic region as reported by the Gistic score. P-Values were computed using Pearson correlation analysis. **C.** Heat map showing the CNV status (left) and the normalized level of expression (right) of 38 genes coding for glycolytic enzymes in 44 PDA cell lines from CCLE. **D.** Dot plot reporting the percentage of 13C glucose incorporation in Malate and Citrate in the two groups of PDA cell lines characterized by the gain/amplification (Amp/Gain) or diploid/deletion (Dipl/Del) of chr12p13 genomic region. **E.** Bar plots showing the distribution of PDA glycolytic subtypes among different molecular subtypes.

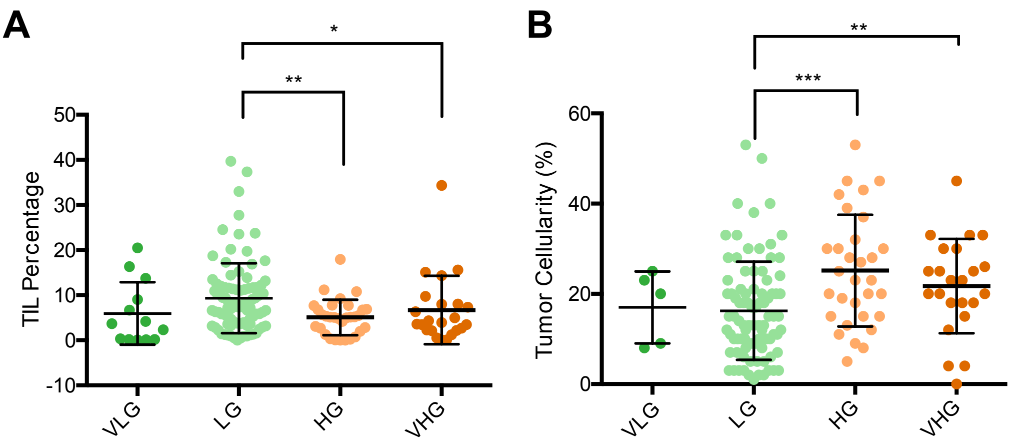

Figure S4. **A.** Dot plot reporting the percentage of Tumor-Infiltrating Lymphocytes (TIL) measured by machine-learning analysis of histological samples of tumors from the TCGA PAAD cohort Saltz et al. (2018). P-Values were computed using Wilcoxon Rank-Sum test. **= p-value <0.01, *= p-value <0.05. **B.** Dot plot reporting the tumor cellularity computed for the tumors from the TCGA PAAD cohort. P-Values were computed using Wilcoxon Rank-Sum test. ***= p-value <0.001, **= p-value <0.01.
